# The association between type 2 diabetes and anhedonic subtype of major depression in hypertensive individuals

**DOI:** 10.1111/jch.14411

**Published:** 2022-01-13

**Authors:** Hadrien Willame, Benjamin Wacquier, Camille Point, Marjorie Dosogne, Mohammed Al Faker, Gwenolé Loas, Matthieu Hein

**Affiliations:** ^1^ Department of Psychiatry and Sleep Laboratory Erasme Hospital Université libre de Bruxelles, ULB Brussels Belgium

**Keywords:** diabetes, epidemiology, hypertension, general, major depression, risk assessment

## Abstract

Given the limited data in the literature, the aim of this study was to investigate the association between type 2 diabetes and anhedonic subtype of major depression in hypertensive individuals. Demographic and polysomnographic data from 323 hypertensive individuals recruited from the database of the Erasme Hospital Sleep Laboratory were analysed. Only individuals with a diagnosis of type 2 diabetes according to the diagnostic criteria of the *American Diabetes Association* at admission were included in the “diabetes group”. Logistic regression analyses were used to study the association between type 2 diabetes and anhedonic subtype of major depression in hypertensive individuals. The rate of type 2 diabetes was 18.9% in our sample of hypertensive individuals. After adjusting for major confounding factors, multivariate logistic regression analyses demonstrated that unlike the non‐anhedonic subtype of major depression, only the anhedonic subtype of major depression was significantly associated with higher likelihood of having type 2 diabetes in hypertensive individuals. In this study, the authors demonstrated that the anhedonic subtype of major depression is significantly associated with type 2 diabetes in hypertensive individuals, which could potentially open up new perspectives for the development of therapeutic strategies complementary to conventional treatments for type 2 diabetes in this subpopulation at high risk of complications related to the co‐occurrence of this metabolic disorder.

## 1 INTRODUCTION

In the literature, there are many arguments for a special relationship between type 2 diabetes and hypertension. Indeed, the prevalence of hypertension may reach 56.7% in type 2 diabetics and that of type 2 diabetes is estimated at 38.7% in hypertensive individuals.^1^, ^2^ In addition, hypertension is a risk factor for type 2 diabetes whereas type 2 diabetes is associated with an increased risk of hypertension.^3^, ^4^ Although this particular relationship is not yet fully understood, the presence of some common pathophysiological mechanisms (deregulation of the renin‐angiotensin‐aldosterone system, oxidative stress, activation of pro‐inflammatory mechanisms, and occurrence of dysfunctional innate/adaptive immune responses) could explain this frequent co‐occurrence of type 2 diabetes and hypertension.^5^ Moreover, the co‐occurrence of these two pathologies is associated with negative impact on life quality and cardiovascular outcome (development of resistance to antihypertensive drugs, increased risk of cardiovascular diseases and higher cardiovascular mortality).^6^, ^7^ Given these various elements, the occurrence of type 2 diabetes in hypertensive individuals is therefore a major public health problem, which justifies the realisation of additional investigations to identify the potential factor associated with type 2 diabetes in hypertension.

In the literature, it has been shown that major depression (MD) may promote the development of type 2 diabetes.^8^ Nonetheless, this particular relationship between MD and type 2 diabetes appears to be mediated by some specific depressive symptoms.^9^ Among the depressive symptoms implicated in this association of MD with type 2 diabetes,^10^ anhedonia is a central symptom of MD that may allow to categorise this psychiatric disorder into two distinct subtypes according to anhedonic status both in the general population and in subpopulations with cardiovascular diseases: the non‐anhedonic subtype of MD (characterised by the maintenance of the ability to feel positive emotions during pleasant life situations) and the anhedonic subtype of MD (characterised by the loss of the ability to feel positive emotions during life situations previously considered to be pleasant).^11^, ^12^ However, despite a significant prevalence of anhedonia complaints in hypertensive individuals,^13^ no study has currently investigated the association between type 2 diabetes and anhedonic subtype of MD in hypertension. Thus, given this lack of validated data in the literature, it would be interesting to study the association between type 2 diabetes and anhedonic subtype of MD in hypertensive individuals to allow a better understanding of the frequent co‐occurrence of hypertension and type 2 diabetes.

The objective of this study was therefore to empirically investigate the association between type 2 diabetes and anhedonic subtype of MD in a large sample of hypertensive individuals. The goal of this approach was to provide healthcare professionals caring for hypertensive individuals with reliable data regarding the association between type 2 diabetes and anhedonic subtype of MD in order to allow the establishment of more targeted therapeutic strategies and better prevention of the negative consequences related to the co‐occurrence of type 2 diabetes in this particular subpopulation.

## 2 MATERIAL AND METHOD

### 2.1 Population

Three hundred and twenty three hypertensive individuals were recruited from the clinical database of the Erasme Hospital Sleep Laboratory, which contains the data of 3301 individuals who usually stayed at the Erasme Hospital Sleep Laboratory between 2017 and 2019. In our study, we did not recruit individuals without hypertension because our objective was to focus on the subpopulation of hypertensive individuals where the co‐occurrence of type 2 diabetes may have a deleterious impact on life quality and cardiovascular outcome.

These hypertensive individuals were referred to the Sleep Laboratory by physicians specialised in sleep medicine after an outpatient consultation during which a preliminary assessment of their complaints related to sleep, their ongoing psychotropic/somatic treatments and their somatic/psychiatric comorbidities was systematically carried out in order to allow a first diagnostic hypothesis. These polysomnographic examinations were performed in these hypertensive individuals to allow an objective assessment of their sleep complaints and exclude the presence of comorbid sleep disorders negatively impacting blood pressure regulation.

The inclusion criteria were age ≥18 years and the presence of hypertension meeting the diagnostic criteria of the *World Health Organisation*.^14^

The exclusion criteria were the presence of diabetes other than type 2 diabetes (such as type 1 diabetes, gestational diabetes, latent autoimmune diabetes, maturity‐onset diabetes of the young, and secondary or iatrogenic diabetes), the presence of severe psychiatric pathologies (psychotic or bipolar disorder), the presence of severe uncontrolled somatic pathologies (chronic liver pathologies, chronic pancreatic pathologies, chronic pulmonary pathologies, severe cardiovascular pathologies, severe renal pathologies, autoimmune pathologies, severe endocrine pathologies, severe neurological pathologies and pathologies altering the activity of the hypothalamic‐pituitary‐adrenal axis such as Cushing's syndrome), the presence of inflammatory or infectious diseases, the presence or history of head trauma, the presence or history of central nervous system damage that may affect the respiratory centres, the presence of craniofacial or thoracic malformations, the presence of ongoing pregnancy, the presence of obstructive sleep apnoea syndrome being treated before the sleep laboratory, the presence of predominantly central sleep apnoea syndrome, the presence of central hypersomnia, the presence of parasomnia, and the presence or history of drug addiction.

### 2.2 Medical, psychiatric, and sleep assessment of participants

A review of their medical records and a complete somatic assessment (including blood test, electrocardiogram, day electroencephalogram and urinalysis) were performed in hypertensive individuals included in this study during their admission to the Erasme Hospital Sleep Laboratory in order to allow a systematic diagnosis of their potential somatic pathologies.

Following this somatic assessment, hypertension was defined as present if one of the following criteria were present: biologically documented self‐reported diagnosis of hypertension; taking anti‐hypertensive medication; mean systolic blood pressure ≥140 mm Hg; mean diastolic blood pressure ≥90 mm Hg. Systolic and diastolic blood pressures were manually measured at the right arm after 5 min of rest in a sitting position by well‐trained nurses. For individuals with a systolic blood pressure ≥140 mm Hg and/or a diastolic blood pressure ≥90 mm Hg, blood pressures were again measured twice after a systematic rest period of five additional minutes. The first measurement was excluded whereas the second and third measurements were averaged in order to minimize the impact of white coat effect. In the absence of prior diagnosis of hypertension, pathological blood pressures were confirmed by repeated measurements during the stay at the sleep laboratory.^14^

Type 2 diabetes was considered as present when the patient at admission has reported a biologically documented diagnosis of type 2 diabetes previously established by a diabetologist according to the diagnostic criteria of the *American Diabetes Association*.^15^ Patients were also assessed as type 2 diabetics when one or more of the following criteria was present at their admission: glycated haemoglobin (HbA1c) ≥6.5%; fasting plasma glucose ≥126 mg/dl (fasting is defined as no calorie intake for at least 8 h); 2‐h plasma glucose ≥200 mg/dl during an oral glucose tolerance test; plasma glucose ≥200 mg/dl in patients with classic symptoms of hyperglycaemia; hyperglycaemic crisis. In the absence of unequivocal hyperglycaemia, criteria 1–3 should be confirmed by repeat testing. In addition, diabetes must have begun in adulthood.^15^

Thereafter, a unit psychiatrist performed a complete psychiatric assessment in hypertensive individuals recruited for this study in order to systematically diagnose their potential psychiatric disorders according to the diagnostic criteria of the DSM 5.^16^ The diagnoses of anhedonic or non‐anhedonic major depressive episodes were therefore made during this systematic psychiatric assessment based on the diagnostic criteria of the DSM 5.^16^ In addition, hypertensive individuals included in this study completed a series of self‐questionnaires to assess the severity of their subjective complaints of depression (Beck Depression Inventory [BDI‐II]), state anhedonia (Anhedonia subscale of Beck Depression Inventory [BDI‐II]), trait anhedonia (Temporal Pleasure Experience Scale), insomnia (Insomnia Severity Index), and daytime sleepiness (Epworth Sleepiness Scale) (detailed description available in Supplementary data ‐ Annex 1).

Finally, in hypertensive individuals recruited for this study, a specific semi‐structured sleep interview and a polysomnographic recording were performed to systematically diagnose their potential comorbid sleep disorders according to the diagnostic criteria of the *American Academy of Sleep Medicine* (detailed description available in Supplementary data ‐ Annex 2).^17^

### 2.3 Statistical analyses

Statistical analyses were performed using Stata 14. The normal distribution of the data was verified using histograms, boxplots, and quantile‐quantile plots whereas the equality of variances was checked using the Levene test.

In order to allow our analyses, we divided our sample of hypertensive individuals into a control group without type 2 diabetes and a patient group with type 2 diabetes. Only hypertensive individuals with a diagnosis of type 2 diabetes according to the diagnostic criteria of the *American Diabetes Association* were included in the “diabetes” group.^15^

Categorical data were described by percentages and numbers whereas continuous variables were described by their median and P25‐P75. Since most continuous data followed an asymmetric distribution, we decided to use non‐parametric tests for all these variables (Wilcoxon test) in order to highlight significant differences between the medians (P25‐P75) observed in the different groups of hypertensive individuals. Finally, the categorical data were described by percentage and were analysed with Chi‐square tests.

Univariate logistic regression models were used to study the association between type 2 diabetes and anhedonic subtype of MD and to identify potential confounding factors (detailed description available in Supplementary Data ‐ Annex 3). In multivariate logistic regression models, the association between type 2 diabetes and anhedonic subtype of MD was only adjusted for significant confounding factors during univariate analyses. These different confounding factors were introduced hierarchically in the different multivariate logistic regression models.

The adequacy of the final model was verified by the Hosmer and Lemeshow test whereas the specificity of the model was verified by the Link test.

The results were considered significant when the *p*‐value was < .05.

## 3 RESULTS

### 3.1 Polysomnographic data (Table 1)

Compared to hypertensive individuals without type 2 diabetes, hypertensive individuals with type 2 diabetes had a reduction in slow‐wave sleep as well as an increase in wake after sleep onset and sleep period time. The two groups did not differ significantly for the other polysomnographic parameters.

**TABLE 1:** Polysomnographic data (*n* = 323)

	Whole sample (*n* = 323)	Hypertensive individuals without diabetes (*n* = 262)	Hypertensive individuals with diabetes (*n* = 61)	*p*‐value
Sleep latency (min)	50.0 (30.0–95.0)	55.5 (30.0–96.0)	39.0 (30.0–75.0)	.115
Sleep efficiency (%)	73.0 (63.0–81.0)	74.0 (63.0–81.0)	72.0 (63.0–80.0)	.479
Sleep period time (min)	441.0 (408.0–471.0)	435.0 (406.0–470.0)	454.0 (429.0–477.0)	.025
Total sleep time (min)	378.0 (321.0–413.0)	378.0 (320.0–414.0)	380.0 (327.0–411.0)	.677
% stage 1	8.0 (6.0–12.0)	8.0 (5.0–12.0)	9.0 (7.0–12.0)	.236
% stage 2	51.0 (43.0–58.0)	50.0 (44.0–58.0)	53.0 (40.0–60.0)	.988
% slow‐wave sleep	7.0 (2.0–13.0)	7.0 (2.0–14.0)	6.0 (1.0–11.0)	.035
% REM latency	15.0 (10.0–19.0)	15.0 (11.0–19.0)	14.0 (8.0–18.0)	.101
REM latency (min)	90.0 (67.0–148.0)	89.5 (67.0–149.0)	91.0 (68.0–137.0)	.721
% wake after sleep onset	12.0 (7.0–22.0)	12.0 (7.0–21.0)	16.0 (10.0–22.0)	.011
Number of awakenings	26 (20–37)	26 (19–36)	27 (22–39)	.100
Micro‐arousal index	15 (9–23)	15 (9–23)	16 (9–27)	.497
Apnoea‐hypopnoea index	13 (4–30)	13 (4–30)	13 (7–28)	.446
Oxygen desaturation index	4 (1–15)	4 (1–15)	7 (2–16)	.251
Total time under 90% of SaO2 (min)	4.0 (.0–32.0)	4.0 (.0–24.0)	8.0 (1.0–62.0)	.066
PLMs index	4 (0–15)	4 (0–15)	3 (0–14)	.284
	Median (P25‐P75)	Median (P25‐P75)	Median (P25‐P75)	Wilcoxon test

*Abbreviations*: PLMs, periodic limb movements during sleep; REM, rapid eye movement.

### 3.2 Demographic data (Table 2)

The rate of type 2 diabetes was 18.9% (*n* = 61) in our sample of hypertensive individuals. Compared to hypertensive individuals without type 2 diabetes, hypertensive individuals with type 2 diabetes had higher body mass index, age, CRP levels and scores on the Anhedonia subscale of the Beck Depression Inventory (items 4, 12 and 21). In addition, body mass index ≥30 kg/m^2^, age ≥65 years, obstructive sleep apnoea syndrome with altered sleep maintenance, complicated hypertension, dyslipidaemia, cardiovascular comorbidities (excluding hypertension), CRP levels ≥1 mg/L and anhedonic subtype of MD were more frequent in hypertensive individuals with type 2 diabetes than in hypertensive individuals without type 2 diabetes. There were no significant differences between the two groups for other demographic parameters. Finally, the rate of non‐anhedonic subtype of MD was 15.5% (*n* = 50) and that of anhedonic subtype of MD was 15.8% (*n* = 51) in our sample of hypertensive individuals.

**TABLE 2:** Sample description (*n* = 323)

Variables		Categories	%	Hypertensive individuals without diabetes	Hypertensive individuals with diabetes	*p*‐value Chi‐square
Sex		Female (*n* = 117)	36.2%	37.0%	32.8%	.535
		Male (*n* = 206)	63.8%	63.0%	67.2%	
BMI (kg/m^2^)		<30 (*n* = 153)	47.4%	51.2%	31.2%	.005
		≥30 (*n* = 170)	52.6%	48.8%	68.8%	
Age (years)		<50 (*n* = 134)	41.5%	42.8%	36.1%	.015
		≥50 & < 65 (*n* = 150)	46.4%	47.7%	41.0%	
		≥65 (*n* = 39)	12.1%	9.5%	22.9%	
Benzodiazepine receptor agonists		No (*n* = 278)	86.1%	84.7%	91.8%	.151
		Yes (*n* = 45)	13.9%	15.3%	8.2%	
Antidepressant therapy		No (*n* = 256)	79.3%	80.5%	73.8%	.241
		Yes (*n* = 67)	20.7%	19.5%	26.2%	
Other psychotropic treatments		No (*n* = 300)	92.9%	93.5%	90.2%	.360
		Yes (*n* = 23)	7.1%	6.5%	9.8%	
Smoking		No (*n* = 256)	79.3%	80.2%	75.4%	.411
		Yes (*n* = 67)	20.7%	19.8%	24.6%	
Alcohol		No (*n* = 146)	45.2%	42.8%	55.7%	.127
		Occasional (*n* = 137)	42.4%	45.0%	31.2%	
		Regular (*n* = 40)	12.4%	12.2%	13.1%	
Snoring		No (*n* = 43)	13.3%	14.1%	9.8%	.375
		Yes (*n* = 280)	86.7%	85.9%	90.2%	
OSAS		No (*n* = 97)	30.0%	31.7%	23.0%	.009
		Without altered sleep maintenance (*n* = 91)	28.2%	30.5%	18.0%	
		With altered sleep maintenance (*n* = 135)	41.8%	37.8%	59.0%	
Insomnia disorders		No (*n* = 115)	35.6%	35.5%	36.0%	.564
		Sleep deprivation alone (n = 86)	26.6%	25.2%	32.8%	
		With sleep duration ≥6 h (*n* = 81)	25.1%	26.3%	19.7%	
		With sleep duration < 6 h (*n* = 41)	12.7%	13.0%	11.5%	
Sleep movement disorders		No (*n* = 243)	75.2%	75.2%	75.4%	.959
		Moderate to severe PLMs alone (*n* = 40)	12.4%	12.6%	11.5%	
		RLS alone or combined with PLMs (*n* = 40)	12.4%	12.2%	13.1%	
Excessive daytime sleepiness		No (*n* = 194)	60.1%	59.5%	62.3%	.693
		Yes (*n* = 129)	39.9%	40.5%	37.7%	
Hypertension status		Untreated (*n* = 153)	47.4%	49.2%	39.3%	.376
		Controlled (*n* = 95)	29.4%	28.2%	34.4%	
		Uncontrolled (n = 75)	23.2%	22.6%	26.3%	
Complicated hypertension		No (*n* = 253)	78.4%	82.1%	62.3%	.001
		Yes (*n* = 70)	21.6%	17.9%	37.7%	
Dyslipidemia		No (*n* = 138)	42.7%	46.2%	27.9%	.009
		Yes (*n* = 185)	57.3%	53.8%	72.1%	
Cardiovascular comorbidities		No (*n* = 261)	80.8%	84.4%	65.6%	.001
		Yes (*n* = 62)	19.2%	15.6%	34.4%	
CRP (mg/L)		<1 (*n* = 85)	26.3%	29.0%	14.8%	.023
		≥1 (*n* = 238)	73.7%	71.0%	85.2%	
Major depression		No (*n* = 222)	68.7%	70.2%	62.3%	.040
		Non‐anhedonic subtype (*n* = 50)	15.5%	16.4%	11.5%	
		Anhedonic subtype (*n* = 51)	15.8%	13.4%	26.3%	
Type 2 diabetes		No (*n* = 262)	81.1%			
		Yes (*n* = 61)	18.9%			

	Median (P25‐P75)			Wilcoxon test
BMI (kg/m^2^)	30.5 (26.8–35.2)	29.8 (26.7–35.0)	33.1 (28.4–36.4)	.004
Age (years)	52 (44–60)	52 (42–59)	55 (48–63)	.006
Systolic blood pressure (mm Hg)	140 (130–145)	140 (130–145)	140 (120–150)	.961
Diastolic blood pressure (mm Hg)	80 (70–90)	80 (70–90)	80 (70–90)	.248
ESS	9 (5–13)	9 (5–13)	9 (5–12)	.664
BDI	10 (5–17)	10 (5–16)	10 (6–21)	.248
BDI–anhedonia	1 (0–3)	1 (0–3)	2 (1–4)	.018
ISI	13 (8–17)	13 (8–17)	12 (8–15)	.160
TEPS	77 (69–84)	77 (69–84)	74 (67–80)	.058
CRP (mg/L)	2.1 (0.9–4.4)	1.9 (0.9–4.0)	3.0 (1.4–5.5)	.022

*Abbreviations*: BMI, body mass index; OSAS, obstructive sleep apnoea syndrome; CRP, C‐Reactive Protein; PLMs, periodic limb movements during sleep; RLS, restless legs syndrome; ESS, Epworth sleepiness scale; BDI, Beck depression inventory; ISI, insomnia severity index; TEPS, temporal experience of pleasure scale.

### 3.3 Univariate regression analyses (Table 3)

Obesity, age ≥65 years, obstructive sleep apnoea syndrome with altered sleep maintenance, complicated hypertension, dyslipidaemia, cardiovascular comorbidities (excluding hypertension), CRP ≥1 mg/L and anhedonic subtype of MD were significantly associated with higher likelihood of having type 2 diabetes in hypertensive individuals.

**TABLE 3:** Univariate analyses (*n* = 323)

Variables	Hypertensive individuals without diabetes	Hypertensive individuals with diabetes	OR (CI 95%)	*p*‐value
Sex				.536
Female	82.9%	17.1%	1	
Male	80.1%	19.9%	1.21 (0.67 to 2.18)	
BMI (kg/m^2^)				.006
<30	87.6%	12.4%	1	
≥30	75.3%	24.7%	2.31 (1.28 to 4.19)	
Age (years)				.020
<50	83.6%	16.4%	1	
≥50 & < 65	83.3%	16.7%	1.02 (0.54 to 1.91)	
≥65	64.1%	35.9%	2.85 (1.28 to 6.33)	
Benzodiazepine receptor agonists				.158
No	79.9%	20.1%	1	
Yes	88.9%	11.1%	0.50 (0.19 to 1.31)	
Antidepressant therapy				.243
No	82.4%	17.6%	1	
Yes	76.1%	23.9%	1.47 (0.77 to 2.81)	
Other psychotropic treatments				.363
Non	81.7%	18.3%	1	
Yes	73.9%	26.1%	1.57 (0.59 to 4.17)	
Smoking				.412
No	82.0%	18.0%	1	
Yes	77.6%	22.4%	1.32 (0.68 to 2.54)	
Alcohol				.132
No	76.7%	23.3%	1	
Occasional	86.1%	13.9%	0.53 (0.29 to 0.98)	
Regular	80.0%	20.0%	0.82 (0.35 to 1.96)	
Snoring				.378
No	86.0%	14.0%	1	
Yes	80.4%	19.6%	1.51 (0.61 to 3.75)	
OSAS				.011
No	85.6%	14.4%	1	
Without altered sleep maintenance	87.9%	12.1%	0.82 (0.35 to 1.90)	
With altered sleep maintenance	73.3%	26.7%	2.16 (1.09 to 4.27)	
Insomnia disorders				.568
No	80.9%	19.1%	1	
Sleep deprivation alone	76.4%	23.6%	1.28 (0.65 to 2.54)	
With sleep duration ≥6 h	85.2%	14.8%	0.74 (0.34 to 1.59)	
With sleep duration < 6 h	82.9%	17.1%	0.87 (0.34 to 2.22)	
Sleep movement disorders				.959
No	81.1%	18.9%	1	
Moderate to severe PLMs	82.5%	17.5%	0.91 (0.38 to 2.18)	
RLS alone or combined with PLMs	80.0%	20.0%	1.07 (0.46 to 2.48)	
Excessive daytime sleepiness				.693
No	80.4%	19.6%	1	
Yes	82.2%	17.8%	0.89 (0.50 to 1.58)	
Hypertension status				.378
Untreated	84.3%	15.7%	1	
Controlled	77.9%	22.1%	1.53 (0.79 to 2.93)	
Uncontrolled	78.7%	21.3%	1.46 (0.72 to 2.95)	
Complicated hypertension				.001
No	85.0%	15.0%	1	
Yes	67.1%	32.9%	2.77 (1.51 to 5.08)	
Dyslipidemia				.010
No	87.7%	12.3%	1	
Yes	76.2%	23.8%	2.22 (1.21 to 4.09)	
Cardiovascular comorbidities				.001
No	84.7%	15.3%	1	
Yes	66.1%	33.9%	2.83 (1.51 to 5.28)	
CRP				.026
<1	89.4%	10.6%	1	
≥1	78.2%	21.8%	2.36 (1.11 to 5.03)	
Major depression				.046
No	82.9%	17.1%	1	
Non‐anhedonic subtype	86.0%	14.0%	0.79 (0.33 to 1.88)	
Anhedonic subtype	68.6%	31.4%	2.21 (1.11 to 4.40)	

*Abbreviations*: BMI, body mass index; OSAS, obstructive sleep apnoea syndrome; CRP, C‐Reactive Protein; PLMs, periodic limb movements during sleep; RLS, restless legs syndrome.

### 3.4 Multivariate regression analyses (Table 4)

After adjustment for the main significant confounding factors during univariate analyses, multivariate logistic regression analyses showed that unlike to the non‐anhedonic subtype of MD, only the anhedonic subtype of MD was significantly associated with higher likelihood of having type 2 diabetes in hypertensive individuals.

**TABLE 4:** Multivariate analyses (*n* = 323)

Variables	Model 1OR adjusted (CI 95%)	*p*‐value	Model 2OR adjusted (CI 95%)	*p*‐value	Model 3 OR adjusted (CI 95%)	*p*‐value	Model 4 OR adjusted (CI 95%)	*p*‐value
Major depression		.018		.031		.040		.040
No	1		1		1		1	
Non‐anhedonic subtype	0.85 (0.34 to 2.08)		0.89 (0.35 to 2.23)		0.95 (0.37 to 2.43)		0.92 (0.36 to 2.37)	
Anhedonic subtype	2.71 (1.31 to 5.63)		2.63 (1.24 to 5.61)		2.61 (1.22 to 5.61)		2.62 (1.21 to 5.66)	

Model 1 = model adjusted for complicated hypertension, dyslipidaemia and cardiovascular comorbidities.

Model 2 = model adjusted for complicated hypertension, dyslipidaemia, cardiovascular comorbidities, age and BMI.

Model 3 = model adjusted for complicated hypertension, dyslipidaemia, cardiovascular comorbidities, age, BMI and OSAS status.

Model 4 = model adjusted for complicated hypertension, dyslipidaemia, cardiovascular comorbidities, age, BMI, OSAS status and CPR levels.

*Abbreviations*: BMI, body mass index; OSAS, obstructive sleep apnoea syndrome; CRP, C‐Reactive Protein.

## 4 DISCUSSION

The rate of type 2 diabetes was 18.9% in our sample of hypertensive individuals. This rate appears to be lower than that of the studies by Marques da Silva and coworkers (2019) (32.9%), Romano and coworkers (2018) (53.0%) and Huang and coworkers (2017) (32.0%), which could be explained by some methodological differences.^18^, ^19^, ^20^ Indeed, unlike our study, the main inclusion criterion in the study by Romano and coworkers (2018) was the presence of resistance to antihypertensive drugs.^19^ However, compared to our study, this recruitment focused only on individuals with hypertension resistant to treatment could have favoured an overestimation of the rate of type 2 diabetes in the study by Romano and coworkers (2018) since resistance to antihypertensive drugs is associated with higher occurrence of type 2 diabetes.^21^ Regarding the studies by Marques da Silva and coworkers (2019) and Huang and coworkers (2017),^18^, ^20^ hypertensive individuals recruited were older than in our study. Indeed, the proportion of hypertensive individuals with an age ≥60 years was higher in the studies by Marques da Silva and coworkers (2019) (72.9%) and Huang and coworkers (2017) (54.7%) than in our study (24.8%). However, given the more frequent occurrence of type 2 diabetes in older individuals,^22^ this higher recruitment of older hypertensive individuals could have led to an overestimation of the rate of type 2 diabetes in the studies by Marques da Silva and coworkers (2019) and Huang and coworkers (2017) compared to our study. Moreover, the rate of type 2 diabetes in our study appears to be higher than that of the study by Tripathy and coworkers (2017) (13.0%),^23^ which could potentially be explained by a underestimation of the rate of type 2 diabetes in this study following the recruitment of a younger population less at risk of type 2 diabetes than that recruited in our study.^24^ Finally, our rate of type 2 diabetes seems to be similar to that of the studies by Bachir Cherif and coworkers (2018) (21.8%), Sever and coworkers (2001) (22.0%) and Lonati and coworkers (2008) (17.5%) where the demographic characteristics of the recruited populations were more similar to those of our sample of hypertensive individuals.^25^, ^26^, ^27^ Thus, regardless of these methodological differences, we have highlighted that type 2 diabetes is a frequent comorbidity in hypertensive individuals, which confirms the importance of strict adherence by healthcare professionals with the guidelines for systematic screening of type 2 diabetes in hypertensive individuals in order to allow better prevention of the potential deleterious consequences related to the co‐occurrence of type 2 diabetes in this particular subpopulation.^28^

In this study, we demonstrated that the rate of anhedonic subtype of MD was 15.8% in our sample of hypertensive individuals, which seems to be consistent with the limited data available in the literature on the frequent occurrence of anhedonia complaints in this particular subpopulation.^29^, ^30^ In addition, similar to some subpopulations,^10^ we demonstrated that unlike to the non‐anhedonic subtype of MD, only the anhedonic subtype of MD was significantly associated with higher likelihood of having type 2 diabetes in hypertensive individuals. Several hypotheses may be formulated in order to better understand this high rate of the anhedonic subtype of MD and its significant association with type 2 diabetes in hypertensive individuals. First, hypertension may promote the development of MD through alterations in brain connectivity induced by several complementary and interconnected pathophysiological mechanisms (such as activation of pro‐inflammatory processes, abnormalities of cerebral perfusion and occurrence of focal vascular white matter lesions).^31^ However, these alterations in cerebral connectivity (in particular at the level of the fronto‐subcortical circuits) may induce a dysfunction of the reward circuit and alter the cognitive processes related to motivation,^32^, ^33^ which could explain the high rate of the anhedonic subtype of MD in hypertensive individuals. Second, in our multivariate regression analyses, we highlighted that the different adjustments performed for the main confounding factors associated with type 2 diabetes (Table 4) had only a limited effect on the higher likelihood of having type 2 diabetes associated with the anhedonic subtype of MD demonstrated in our sample of hypertensive individuals, which seems to indicate that in this particular subpopulation, this higher likelihood of having type 2 diabetes associated with the anhedonic subtype of MD could be mainly mediated by pathophysiologic mechanisms specific to anhedonia that occur independently of these main confounding factors. Indeed, in major depressed individuals, anhedonia seems to favour a higher consumption of “comfort” foods characterised by high levels of refined sugar and saturated fat, which could correspond to an attempt at self‐medication to increase the hedonic tone.^34^, ^35^ In addition, alongside these dietary changes, anhedonia also appears to be associated with more sedentary lifestyle characterised by decreased physical activity.^36^ However, the occurrence of these deleterious behavioural changes related to anhedonia (combination of high‐calorie diet and more marked sedentary behaviours) may directly promote the development of insulin resistance that plays a central role in the pathophysiology of type 2 diabetes.^37^ Thus, following these different elements, it seems essential to screen more systematically and adequately treat the anhedonic subtype of MD in hypertensive individuals in order to allow better management of type 2 diabetes in this subpopulation at high risk of complications related to the co‐occurrence of this metabolic disorder.

Although there is evidence in the literature for a beneficial effect of some conventional treatments for MD on glycaemic control in major depressed individuals with type 2 diabetes,^38^ the higher likelihood of having type 2 diabetes associated with the anhedonic subtype of MD demonstrated in our study could promote the development of new therapies complementary to conventional glycaemic control strategies in major depressed hypertensive individuals with type 2 diabetes. Indeed, since the anhedonic subtype of MD appears to have a negative effect on glycaemic control in major depressed individuals with type 2 diabetes,^39^ it could be interesting to implement more targeted therapeutic strategies in anhedonic major depressed hypertensive individuals with type 2 diabetes. Among the pharmacological therapies targeting anhedonia, the use alone or in combination of bupropion (targeting the reward circuit through modulation of the noradrenergic and dopaminergic systems) seems to show promising results on both depressive symptoms and glycaemic control in major depressed individuals with type 2 diabetes.^40^, ^41^ However, one of the rare side effects of bupropion is the occurrence of hypertensive crises, which justifies strict compliance with the recommendations for the use of this molecule in the particular subpopulation of major depressed hypertensive individuals.^42^ Regarding non‐pharmacological approaches targeting anhedonia, despite the great heterogeneity of the techniques available in the literature, some "well‐being" interventions (such as reinforcing positive affects) appear to be associated with improved depressive symptoms and better glycaemic control in major depressed individuals with type 2 diabetes.^43^ However, despite this potential positive effect on glycaemic control of therapies targeting anhedonia, these therapeutic strategies should only be considered in addition to adequate combined treatment of type 2 diabetes and hypertension in anhedonic major depressed hypertensive individuals with type 2 diabetes. Indeed, given the negative impact of the co‐occurrence of type 2 diabetes and hypertension on glycaemic control, it is essential to respect the guidelines for the combined treatment of type 2 diabetes and hypertension in order to allow optimal glycaemic control in individuals with hypertension and type 2 diabetes.^44^, ^45^, ^46^, ^47^ Regarding the treatment of type 2 diabetes in hypertensive individuals, it is currently recommended to implement lifestyle modifications alone or combined with pharmacological treatment (oral glucose‐lowering drugs and/or insulin therapy) depending on the initial severity of type 2 diabetes, the objective of glycaemic control and potential comorbid conditions.^44^, ^45^ On the other hand, regarding the treatment of hypertension in type 2 diabetics, the establishment of lifestyle modifications combined with antihypertensive therapy is currently recommended in order to allow optimal blood pressure control (< 130/80 mm Hg) in type 2 diabetics with hypertension.^46^, ^47^ However, in this particular subpopulation, the use alone or in combination of antihypertensive drugs with a demonstrated positive impact on the reduction of cardiovascular mortality (angiotensin‐converting‐enzyme inhibitors, angiotensin receptor blockers, thiazide‐like diuretics and dihydropyridine calcium channel blockers) should be preferred based on the initial severity of hypertension, the goal of blood pressure control and potential comorbid conditions.^46^, ^47^ Thus, given the potential positive effect of therapies targeting anhedonia on glycaemic control, the implementation of these therapies targeting anhedonia in addition to an adequate combined treatment of type 2 diabetes and hypertension could be a promising therapeutic option to potentiate conventional glycaemic control strategies in anhedonic major depressed hypertensive individuals with type 2 diabetes.

### 4.1 Limitations

The results obtained in our study come from retrospective data that, even if they have been encoded in a systematic manner, cannot be verified directly with the patient in most cases, which means that our results need to be replicated in prospective studies. Furthermore, since other types of diabetes were exclusion criteria in our study, our results are only applicable to type 2 diabetes, which may possibly limit their generalisation. In addition, we only focused on the anhedonic and non‐anhedonic subtypes of MD, which means that our results cannot be generalised to other subtypes of MD. Finally, our database only contains hypertensive individuals who have agreed to perform a sleep laboratory, which may also limit the generalisation of our results.

## 5 CONCLUSIONS

In our sample of hypertensive individuals, the prevalence of type 2 diabetes was 18.9%, which seems to confirm that type 2 diabetes is a frequent comorbidity in hypertension. In addition, we have shown that unlike the non‐anhedonic subtype of MD, only the anhedonic subtype of MD was significantly associated with higher likelihood of having type 2 diabetes in hypertensive individuals, which could potentially open up new perspectives for the development of therapeutic strategies complementary to conventional treatments for type 2 diabetes in this subpopulation at high risk of complications related to the co‐occurrence of this metabolic disorder.

## CONFLICT OF INTEREST

The authors have no conflicts of interest with the work carried out in this study.

## Supporting information

SUPPORTING INFORMATION
