# Supplementary material for: The association between type 2 diabetes and anhedonic subtype of major depression in hypertensive individuals
Source: J Clin Hypertens (Greenwich). 2022 Jan 13;24(2):156–66. doi: 10.1111/jch.14411 (PMC8845468; doi:10.1111/jch.14411)
Supplement: Supplementary file 1 — SUPPORTING INFORMATION [file JCH-24-156-s001.docx]

# Supplementary data

# Annex 1

Detailed description of self-questionnaires used

- The presence of depressive symptoms was investigated using Beck Depression Inventory (BDI-II). This scale consists of 21 items that may be scored from 1 to 3. The score may vary from 0 to 63. A score of 0-9 indicates no depression, 10-18 mild depression, 19-29 moderate depression, and 30-63 severe depression.^1^
- State anhedonia (recent change) was studied using the Anhedonia subscale of Beck Depression Inventory (BDI-II) that contains three items (4, 12 and 21). This subscale of state anhedonia was validated by Joiner et al. (2003).^2^
- Trait anhedonia (stable over time) was assessed using the Temporal Pleasure Experience Scale (TEPS). TEPS is an 18-items questionnaire: 10 items measure anticipatory pleasure (anticipatory pleasure subscale [TEPS-ANT]) and 8 items measure consuming pleasure (consuming pleasure subscale [TEPS-CONS]). For each item, the subjects respond with a severity gradient ranging from 1 (completely false) to 6 (very true). The score of the anticipatory pleasure subscale varies between 10 and 60 and that of consuming pleasure between 8 and 48. The subject must describe himself as he usually is, since it is an assessment of trait anhedonia and not of state anhedonia.^3^
- Daytime sleepiness was investigated using the Epworth Sleepiness Scale. This scale consists of 8 questions that may be scored from 0 to 3 and assesses daytime sleepiness in frequent situations of daily life. The score may vary from 0 to 24. A score greater than 10 indicates excessive daytime sleepiness.^4^
- The presence of insomnia symptoms was investigated using the Insomnia Severity Index. This index consists of 7 questions that may be scored from 0 to 4. The score may vary from 0 to 28. A score of 0-7 indicates no insomnia, 8-14 subclinical insomnia, 15-21 moderate insomnia, and 22-28 severe insomnia.^5^

**References**

1/Beck AT, Steer RA, Ball R, Ranieri W. Comparison of Beck Depression Inventories -IA and -II in psychiatric outpatients. J Pers Assess. 1996;67(3):588-597.

2/Joiner TE, Brown JS, Metalsky GI. A test of the tripartite model's prediction of anhedonia's specificity to depression: patients with major depression versus patients with schizophrenia. Psychiatry Res. 2003;119(3):243-250.

3/Loas G, Monestes J-L, Ameller A, Bubrovszky M, Yon V, Wallier J, Berthoz S, Corcos M, Thomas P, Gard DE. Traduction et étude de validation de la version française de l’échelle d’expérience temporelle du plaisir (EETP, Temporal Experience of Pleasure Scale [TEPS], Gard et al., 2006): étude chez 125 étudiants et chez 162 sujets présentant un trouble psychiatrique. Ann. Méd. Psychol. 2009;167:641–648.

4/Johns MW. A new method for measuring daytime sleepiness: the Epworth sleepiness scale. Sleep. 191;14(6):540-545.

5/Morin CM. Insomnia: psychological assessment and management. Guilford Press, New York: 1993.

**Annex 2**

Sleep assessment of participants

In hypertensive individuals recruited for this study, a specific semi-structured sleep interview based on the recommendations of the *American Academy of Sleep Medicine* was performed by a unit psychiatrist during their admission to the Sleep Laboratory in order to allow a systematic assessment of their complaints related to sleep including sleeping habits, symptoms of insomnia disorder, symptoms of sleep-related breathing disorders, symptoms of central disorders of hypersomnolence, symptoms of circadian rhythm sleep-wake disorders, symptoms of parasomnias and symptoms of sleep-related movement disorders.^1^ This specific semi-structured sleep interview is a standardised procedure of the Erasme Hospital Sleep Laboratory that makes it possible to systematically research for symptoms suggestive of sleep disorders and to program polysomnographic recordings adapted to the symptoms reported by patients.

During their stay in the Sleep Laboratory, hypertensive individuals included in this study benefited from a polysomnographic recording from which the data were collected for analysis. The patients went to bed between 22:00 - 24:00 and got up between 6:00 - 8:00, following their usual schedule. During bedtime hours, the subjects were recumbent and the lights were turned off. Daytime naps were not permitted.

The polysomnographic recordings performed in our unit meet the recommendations of the *American Academy of Sleep Medicine*.^2^ The applied polysomnography-montage was as follows: two electro-oculogram channels, three electroencephalogram channels (Fz-Ax, Cz-Ax, and Oz-Ax, where Ax was a A1A2 mastoid reference), one submental electromyogram channel, electrocardiogram, pressure cannula to detect the oro-nasal airflow, finger pulse-oximetry, a microphone to record breathing sounds and snoring, plethysmographic inductive belts to measure thoracic and abdominal breathing, and anterior tibialis electrodes. Polysomnographic recordings were visually scored by specialised technicians according to the criteria of the *American Academy of Sleep Medicine*.^3^

Obstructive apnoeas were scored if the decrease in air flow was ≥90% for at least 10 seconds whereas obstructive hypopnoeas were scored if the decrease in airflow was ≥30% for at least 10 seconds with a decrease in oxygen saturation of 3% or followed by microarousal.^4^ The obstructive apnoea-hypopnoea index correspond to the total number of obstructive apnoeas and hypopnoeas divided by the period of sleep in hours. Obstructive sleep apnoea syndrome was considered as present when the obstructive apnoea-hypopnoea index was ≥5/hour.^5^

Periodic limb movements during sleep were scored on the basis of the following strict criteria: 1) duration between 0.5 to 10 seconds, 2) interval between 5 and 90 seconds from leg movement onset and 3) movements had to be part of a series of ≥4 consecutive movements meeting these criteria.^6^ Periodic limb movement’s index corresponds to the total number of periodic limb movements during sleep divided by period of sleep in hours. Moderate to severe periodic limb movements during sleep were considered to be present when the periodic limb movement’s index was ≥15/hour.^7^ Moreover, the diagnoses of restless legs syndrome were made according to the diagnostic criteria of the *International Restless Legs Syndrome Study Group*.^8^

Finally, potential diagnoses of insomnia disorders were made according to the diagnostic criteria of the *American Academy of Sleep Medicine Work Group*^9^ whereas short sleep duration was defined as sleep time <6 hours.^10^

**References**

1/American Academy of Sleep Medicine. International Classification of Sleep Disorders (3rd ed.) Darien, IL:2014.

2/Kushida CA, Littner MR, Morgenthaler T, Alessi CA, Bailey D, Coleman J Jr, Friedman L, Hirshkowitz M, Kapen S, Kramer M, Lee-Chiong T, Loube DL, Owens J, Pancer JP, Wise M. Practice parameters for the indications for polysomnography and related procedures: an update for 2005. Sleep. 2005;28(4):499-521.

3/Iber C, Ancoli-Israel S, Chesson A, Quan SF for the American Academy of Sleep Medicine. The AASM Manual for the Scoring of Sleep and Associated Events: Rules, Terminology and Technical Specifications. 1st. ed: American Academy of Sleep Medicine, Westchester, IL;2007.

4/Berry RB, Budhiraja R, Gottlieb DJ, Gozal D, Iber C, Kapur VK, Marcus CL, Mehra R, Parthasarathy S, Quan SF, Redline S, Strohl KP, Davidson Ward SL, Tangredi MM; American Academy of Sleep Medicine. Rules for scoring respiratory events in sleep: update of the 2007 AASM Manual for the Scoring of Sleep and Associated Events. Deliberations of the Sleep Apnea Definitions Task Force of the American Academy of Sleep Medicine. J Clin Sleep Med. 2012;8(5):597-619.

# 5/Fleetham J, Ayas N, Bradley D, Ferguson K, Fitzpatrick M, George C, Hanly P, Hill F, Kimoff J, Kryger M, Morrison D, Series F, Tsai W; CTS Sleep Disordered Breathing Committee. Canadian Thoracic Society guidelines: diagnosis and treatment of sleep disordered breathing in adults. Can Respir J. 2006;13(7):387-392.

# 6/Ferri R, Koo BB, Picchietti DL, Fulda S. Periodic leg movements during sleep: phenotype, neurophysiology, and clinical significance. Sleep Med. 2017;31:29-38.

# 7/Haba-Rubio J, Marti-Soler H, Tobback N, Andries D, Marques-Vidal P, Vollenweider P, Preisig M, Heinzer R. Clinical significance of periodic limb movements during sleep: the HypnoLaus study. Sleep Med. 2018;41:45-50.

# 8/Allen RP, Picchietti DL, Garcia-Borreguero D, Ondo WG, Walters AS, Winkelman JW, Zucconi M, Ferri R, Trenkwalder C, Lee HB; International Restless Legs Syndrome Study Group. Restless legs syndrome/Willis-Ekbom disease diagnostic criteria: updated International Restless Legs Syndrome Study Group (IRLSSG) consensus criteria--history, rationale, description, and significance. Sleep Med. 2014;15(8):860-873.

9/Edinger JD, Bonnet MH, Bootzin RR, Doghramji K, Dorsey CM, Espie CA, Jamieson AO, McCall WV, Morin CM, Stepanski EJ; American Academy of Sleep Medicine Work Group. Derivation of research diagnostic criteria for insomnia: report of an American Academy of Sleep Medicine Work Group. Sleep. 2004;27(8):1567-1596.

10/Hein M, Lanquart JP, Loas G, Hubain P, Linkowski P. Insomnia with short sleep duration as risk factor for type 2 diabetes: a systematic review of the literature. Rev Med Brux. 2020;41(2):98-104.

**Annex 3**

Description of the confounding factors included in univariate analyses

After a review of the literature on factors associated with type 2 diabetes,^1,2^ the potential confounding factors included in this study were body mass index (categorised: <30 kg/m², ≥30 kg/m²), age (categorised: <50 years, ≥50 & <65 years, ≥65 years), alcohol consumption (categorised: no, occasional, regular), obstructive sleep apnoea syndrome (categorised: no, obstructive sleep apnoea syndrome without altered sleep maintenance, obstructive sleep apnoea syndrome with altered sleep maintenance), insomnia disorders (categorised: no, sleep deprivation alone, with sleep duration <6 hours, with sleep duration ≥6 hours), sleep movement disorders (categorised: no, moderate to severe periodic limb movements during sleep alone, restless leg syndrome alone or combined with periodic limb movements during sleep), hypertension status (categorised: untreated, controlled, uncontrolled), CRP levels (categorised: <1 mg/L, ≥1 mg/L) and as binary variables: gender, benzodiazepine receptor agonists, antidepressant therapy, other psychotropic medications (antipsychotics or thymostabilisers), smoking, snoring, excessive daytime sleepiness, complicated hypertension, cardiovascular comorbidities (excluding hypertension) and dyslipidaemia.

**References**

1/Hein M, Lanquart JP, Loas G, Hubain P, Linkowski P. Prevalence and Risk Factors of Type 2 Diabetes in Major Depression: A Study on 703 Individuals Referred for Sleep Examinations. Psychosomatics. 2018;59(2):144-157.

2/Hein M, Lanquart JP, Loas G, Hubain P, Linkowski P. Prevalence and risk factors of type 2 diabetes in insomnia sufferers: a study on 1311 individuals referred for sleep examinations. Sleep Med. 2018;46:37-45.
